# Circular RNA GRB10 as a competitive endogenous RNA regulating nucleus pulposus cells death in degenerative intervertebral disk

**DOI:** 10.1038/s41419-017-0232-z

**Published:** 2018-02-23

**Authors:** Wei Guo, Bin Zhang, Kun Mu, Shi-Qing Feng, Zhan-Yin Dong, Guang-Zhi Ning, Hao-Ran Li, Shen Liu, Ling Zhao, Yan Li, Bing-Bing Yu, Hui-Quan Duan, Chao Sun, Yong-Jin Li

**Affiliations:** 1 Department of Orthopaedics, Hebei Province Cangzhou Hospital of Integrated Traditional and Western Medicine (Cangzhou No.2 Hospital), 31 Huanghe Road, Hebei 061001 Cangzhou, China; 2 Department of Orthopaedics, Tianjin Medical University General Hospital, 154 Anshan Road, Heping District, 300052 Tianjin, China; 3 Department of Breast Surgery, Hebei Province Cangzhou Hospital of Integrated Traditional and Western Medicine (Cangzhou No.2 Hospital), 31 Huanghe Road, Hebei 061001 Cangzhou, China

## Abstract

Intervertebral disc degeneration (IDD) is an important factor leading to low back pain, but the underlying mechanisms remain poorly understood. Compared with normal nucleus pulposus (NP) tissues, the expression of circ-GRB10 was downregulated in IDD. Furthermore, overexpression of circ-GRB10 inhibited NP cell apoptosis. circ-GRB10 could sequester miR-328-5p, which could potentially lead to the upregulation of target genes related to cell proliferation via the ErbB pathway. In conclusion, the present study revealed that circ-GRB10/miR-328-5p/ERBB2 signaling pathway is involved in IDD development, suggesting that circ-GRB10 might be a novel therapeutic target for IDD.

## Introduction

Intervertebral disc degeneration (IDD) is characterized by extracellular matrix breakdown and abnormal matrix synthesis leading to reduced hydration, loss of disc height, and decreased potential to absorb load^1,2^. It is considered as the predominant cause for chronic low back pain and spine-related disease, leading to economic and social burden worldwide^3^. It is estimated that as much as 84% of the population suffers from low back pain at some point in their lifetime, whereas 10% are chronically disabled^4^. Nevertheless, current strategies for IDD treatment is hampered by an incomplete understanding of its pathogenesis. Nowadays, IDD treatment is limited to symptomatic interventions, which do not adequately improve outcomes since no disease-modifying drugs are available^5^. Consequently, the clinical management of diseases related to IDD remains severely limited. Therefore, unveiling the pathophysiology and molecular mechanisms underlying IDD is imperative for developing novel therapeutic approaches.

The intervertebral disc is the largest avascular structure in the body^6^. Studies have shown that blood vessels do not enter the nucleus pulposus (NP) tissue, and NP cells in the center of an adult disk can be up to 8 mm away from the nearest blood vessel^7^. Accumulating evidence indicated that a variety of cellular events are dysregulated in the progression of IDD, ranging from NP cell apoptosis to cytokine expression^7–12^. Excessive apoptosis of intervertebral disc cells play an essential role in IDD^7^. Both genetic and environmental factors contribute to the development of IDD, but genetic factors are considered to be the most important contributors^13–16^.

Non-coding RNAs (ncRNAs) are a recently discovered, but very important part of the cells’ genetic machinery^17,18^. ncRNAs account for 98% of the human genome devoid of protein-coding function. Among the various classes of ncRNAs, we find circular RNA (circRNA), which are present in all living organisms. Non-coding RNAs play critical roles in a variety of biological processes pertaining to gene expression^19,20^.

Although a number of molecular drivers of IDD have been described over the past years, ncRNAs have emerged recently as key players in the pathogenesis of IDD^21,22^. Circular RNAs act as post-transcriptional regulators and they can interact with microRNAs (miRNAs) via miRNA sponges and competitive endogenous RNA (ceRNA) in the cytoplasm^23–25^. MicroRNA sponges are a circRNA with miRNA-binding sites that could absorb the miRNA and eliminate its repressive action on the miRNA target.

In this study, we performed a comprehensive analysis using bioinformatics, identified several IDD-specific circRNAs, and discovered that the expression of circ-GRB10 was significantly downregulated in degenerative NP tissues compared with normal NP tissues. Subsequently, we systematically validated the role of circ-GRB10 in cultured human NP cells.

## Results

### Identification of differentially expressed circRNAs

**Fig. 1:**
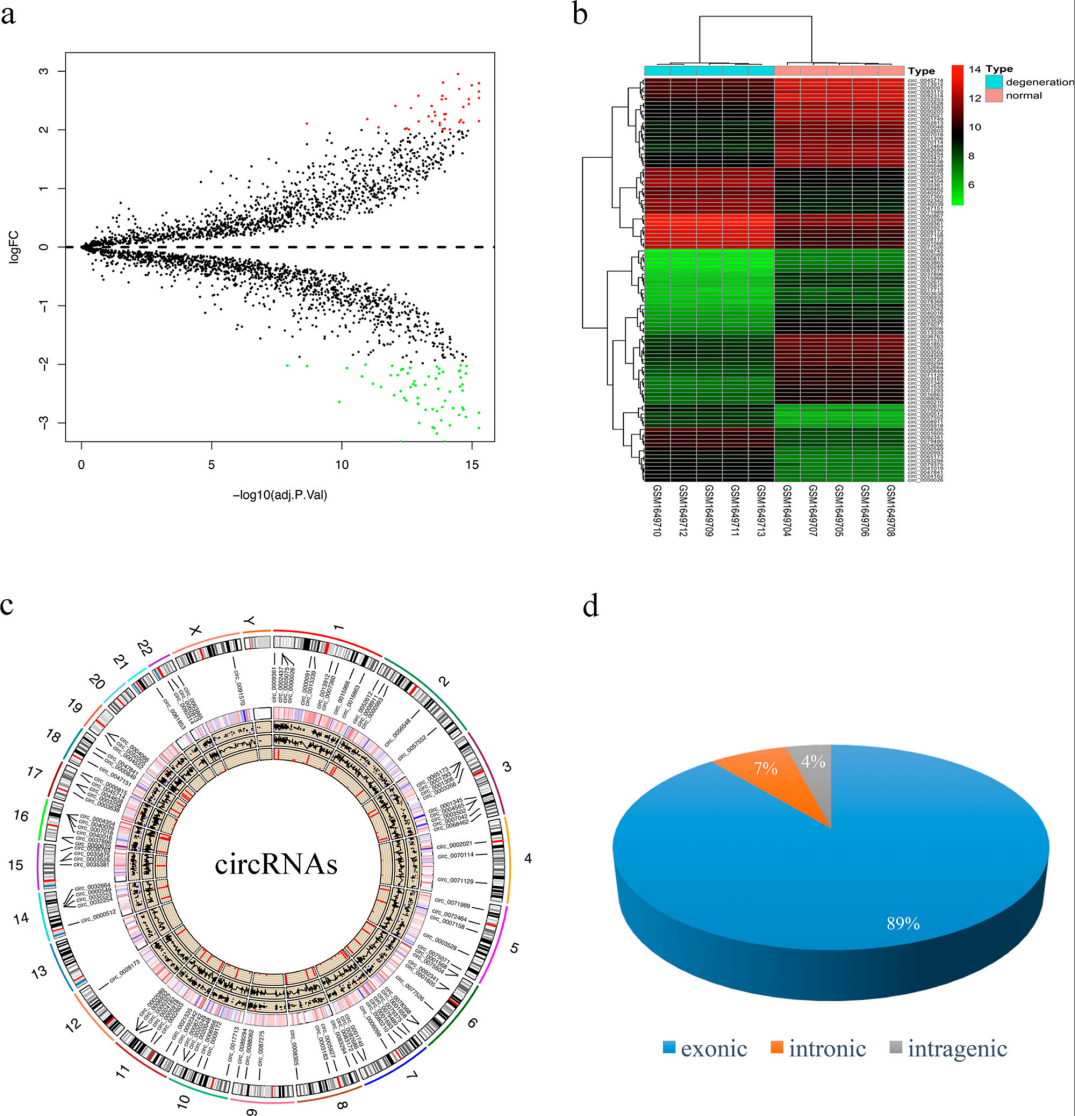
Differences and characterization in circRNA expression profiles between IDD and normal NP tissues. **a** Volcano plots are used for visualizing differential expression between two different conditions. The horizontal lines correspond to 2.0-fold (log2 scaled) up and down, respectively, and the vertical line represents an adjusted *P* value of 0.05 (−log10 scaled). The red and green points represent the upregulated- and downregulated-expressed circRNAs, with statistical significance. **b** Hierarchical cluster analysis of the significantly upregulated and downregulated circRNAs. Each column represents a sample and each row represents a circRNA. Red represents high relative expression and green represents low relative expression. **c** RCircos image showing human chromosome representation with data tracks for connectors, differentially expressed circRNA labels, heatmap of all circRNAs (red and green represent the upregulated and downregulated circRNAs compared with normal NP tissues), scatter plot of all circRNAs, line plot of all circRNAs, and histogram of significantly differential expressed circRNAs. **d** Constituent ratios of differentially expressed circRNAs

After data normalization (Supplementary Figure 1), 104 differentially expressed circRNAs were identified: 41 circRNAs were upregulated while 63 circRNAs were downregulated (Supplementary Table S1). As shown in Fig. 1a, the volcano plot identified significantly differentially regulated circRNAs between the two groups. Hierarchical clustering showed that circRNA expression patterns were distinguishable between IDD and normal control samples (Fig. 1b). The distribution of the circRNAs on the human chromosomes is depicted in Fig. 1c. Among them, 89% of differentially expressed circRNAs are transcribed from protein-coding exons; 7% are from introns; and 4% are from intragenic regions (Fig. 1d). The results suggested that most differentially expressed circRNAs may act as miRNA sponges in degenerative NP cells.

### circRNA–miRNA interaction network

**Fig. 2:**
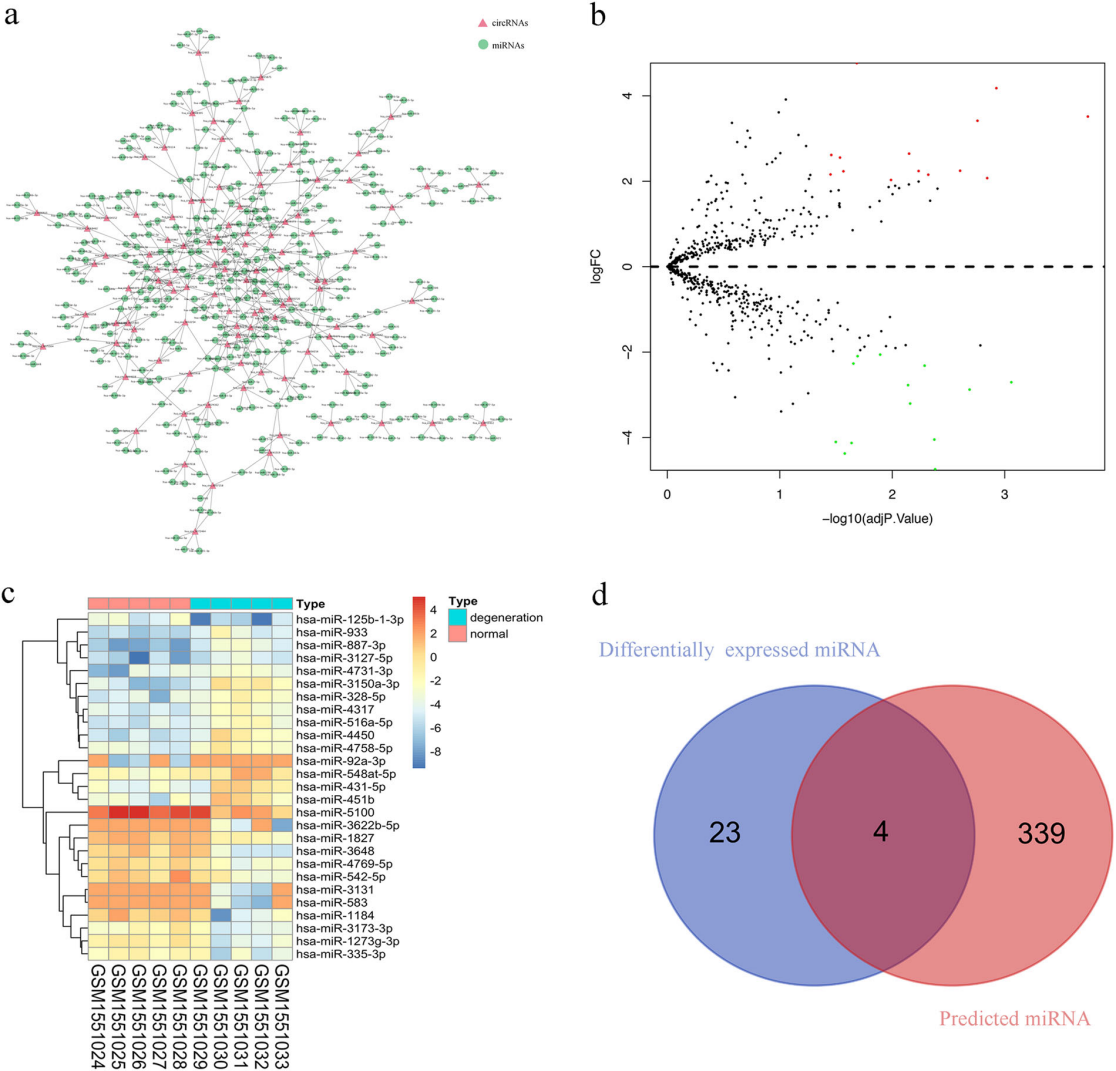
circRNA–miRNA network analysis. **a** The panorama network consists of 104 dysregulated circRNAs and their 343 target miRNAs. **b** Volcano plots are used for visualizing differential expression between two different conditions. The horizontal lines correspond to 2.0-fold (log2 scaled) up and down, respectively, and the vertical line represents an adjusted *P* value of 0.05 (−log10 scaled). The red and green points represent the upregulated- and downregulated-expressed miRNAs with statistical significance. **c** Hierarchical cluster analysis of the significantly upregulated and downregulated miRNAs. Each column represents a sample and each row represents a miRNA. Red strip represents high relative expression and green strip represents low relative expression. **d** Venn diagram demonstrating the intersection of differentially expressed miRNAs and predicted miRNA

To determine the function of circRNAs, interactions between circRNAs and their target miRNAs were theoretically predicted by conserved seed-matching sequence using the TargetScan (http://www.targetscan.org/) and miRanda (http://www.miranda-im.org/) databases. All the differentially expressed circRNAs were predicted according to their complementary miRNA matching sequence. A total of 343 miRNAs were predicted to combine with the 104 circRNAs (Supplementary Table S1). An entire network of circRNA/miRNA interaction was delineated using Cytoscape (Fig. 2a).

The circRNA–miRNA axis in degenerative NP cells was predicted by combining miRNA chip data from the same sample source of circRNA chip. After data normalization (Supplementary Figure 2), 27 differentially expressed miRNAs were screened out from NP cells expression profile: 14 miRNAs were upregulated and 13 miRNAs were downregulated (Supplementary Table S2). The volcano plot identified significantly differentially changed miRNAs between the two groups (Fig. 2b). Hierarchical clustering showed that miRNAs expression patterns were distinguishable between IDD and normal control samples (Fig. 2c). The Venn diagram revealed the intersection of differentially expressed miRNAs and predicted miRNAs (Fig. 2d). hsa-miR-335-3p, hsa-miR-328-5p, hsa-miR-583, and hsa-miR-92a-3p are miRNAs related to 12 circRNAs (Table 1). The Pearson correlation coefficient between the circRNAs and miRNAs was compared (Table 1): hsa_circ_0080210 (circ-GRB10) and hsa-miR-328-5p (miR-328-5p) were significantly negatively correlated (*P* = 0.012).

**Table 1:** The correlations between the circRNAs and miRNAs

Differentially expressed circRNAs	Differentially expressed miRNAs	Pearson’s correlation	*P* value
hsa_circ_0002089	hsa-miR-335-3p	0.69	0.027
hsa_circ_0007360	hsa-miR-335-3p	−0.658	0.039
hsa_circ_0017713	hsa-miR-335-3p	0.595	0.070
hsa_circ_0057552	hsa-miR-335-3p	−0.646	0.044
hsa_circ_0065173	hsa-miR-335-3p	−0.67	0.034
hsa_circ_0047841	hsa-miR-335-3p	−0.661	0.038
hsa_circ_0001293	hsa-miR-328-5p	−0.695	0.018
hsa_circ_0004066	hsa-miR-328-5p	0.728	0.017
hsa_circ_0080210	hsa-miR-328-5p	−0.755	0.012
hsa_circ_0007158	hsa-miR-583	−0.634	0.049
hsa_circ_0005918	hsa-miR-92a-3p	0.662	0.037
hsa_circ_0040016	hsa-miR-92a-3p	−0.634	0.049

### Validation of the differential expression levels and correlation between circ-GRB10 and miR-328-5p

**Fig. 3:**
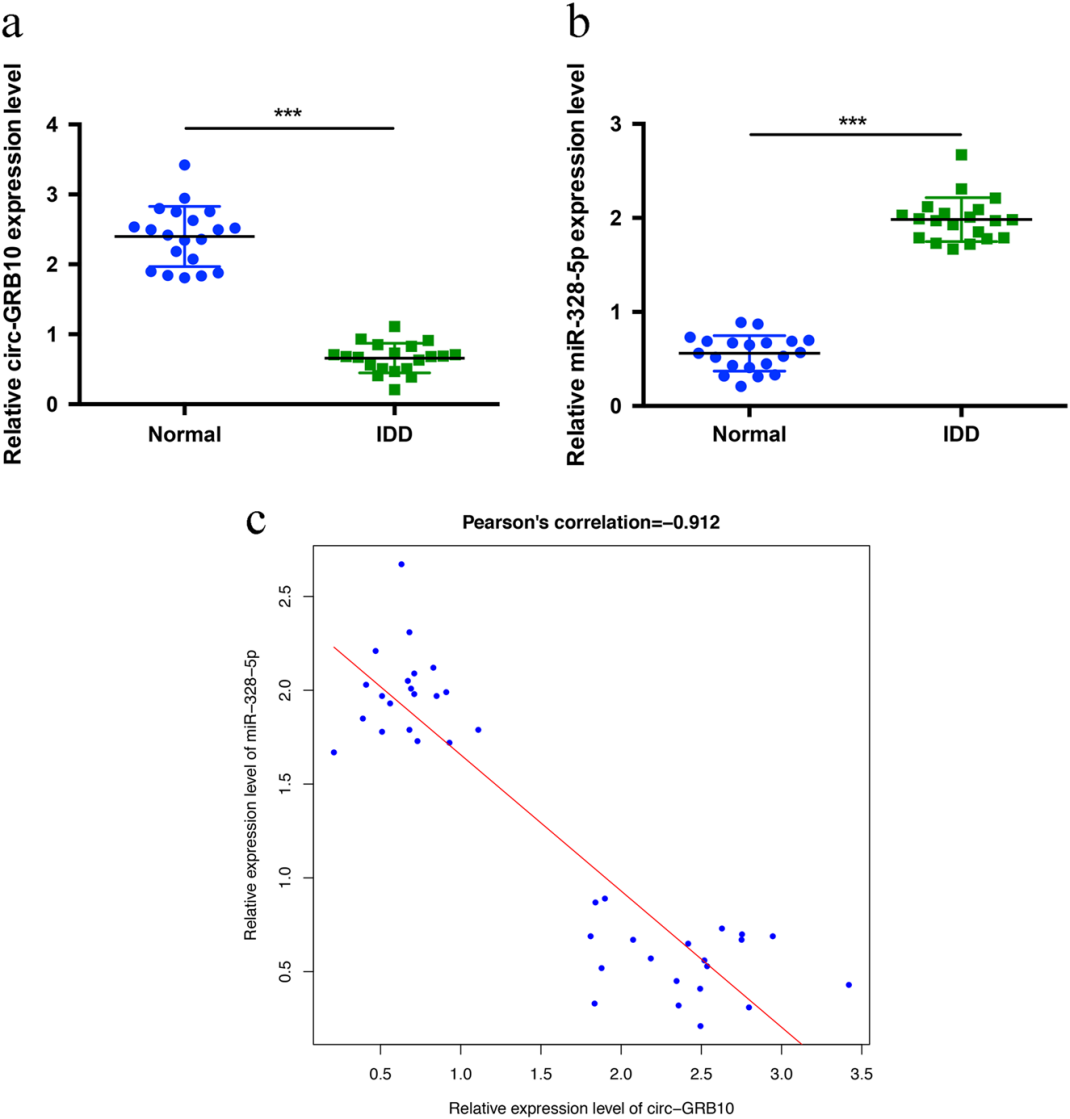
Differential expression levels and the correlation of circ-GRB10 and miR-328-5p. **a** The expression of circ-GRB10 in NP tissues was measured in 20 patients and 20 controls using qRT-PCR (***P < 0.001). **b** The expression of miR-328-5p was significantly upregulated in degenerative NP tissues (***P < 0.001). **c** circ-GRB10 expression was significantly negative correlated with miR-328-5p expression

The circ-GRB10 and miR-328-5p expression levels were validated by qRT-PCR in 40 pairs of samples. circ-GRB10 was significantly downregulated in IDD NP tissues, while the miR-328-5p expression was significantly higher in degenerative NP tissues (Fig. 3a, b). Interestingly, circ-GRB10 and miR-328-5p demonstrated a significant negative correlation (*r* = −0.912, Fig. 3c). Therefore, we hypothesized that circ-GRB10 may function as a miR-328-5p sponge in NP cells.

### Subcellular localization of circ-GRB10 and miR-328-5p

**Fig. 4:**
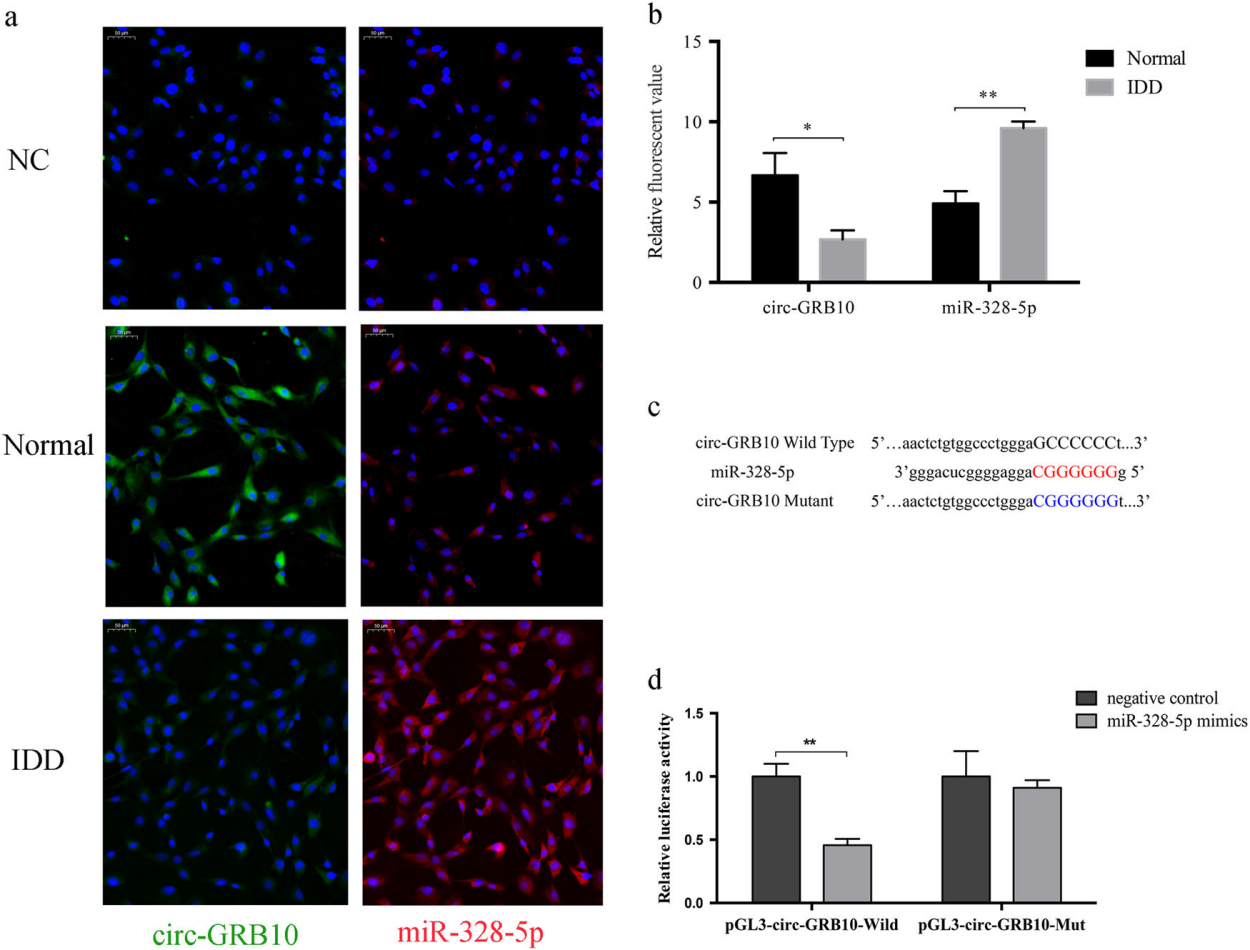
Circ-GRB10 serves as a sponge for miR-328-5p. **a** FISH assay showing that circ-GRB10 was mostly located in cytoplasm of NP cell and so was the miR-328-5p. The negative control (NC) was made of cells treated with RNAse. **b** The immunofluorescence values of circ-GRB10 and miR-328-5p in each group. *P < 0.05, **P < 0.001, *n* = 4. **c** Sequence alignment of human miR-328-5p with circ-GRB10. Bottom: mutations in the circ-GRB10 sequence to create the mutant luciferase reporter constructs. **d** Luciferase reporter assay in NP cells after transfected with negative control or miR-328-5p mimics, renilla luciferase vector pRL-SV40, and the reporter constructs. Both firefly and renilla luciferase activities are measured in the same sample. Firefly luciferase signals were normalized with renilla luciferase signals. **P < 0.001, *n* = 3

Subcellular localization of ncRNAs determines their mode of action. Fluorescence in situ hybridization (FISH) was performed to determine the localization of circ-GRB10 and miR-328-5p expression in NP cells. As expected, circ-GRB10 and miR-328-5p were co-localized in the cytoplasm (Fig. 4a). Moreover, circ-GRB10 was downregulated in degenerative NP cells (*P* < 0.05), while miR-328-5p was upregulated (*P* < 0.01) (Fig. 4b).

### Circ-GRB10 function as a sponge for miR-328-5p in NP cells

As predicted by the TargetScan and miRanda databases, circ-GRB10 was shown to have binding sites for miR-328-5p (Fig. 4c). To validate the interaction between circ-GRB10 and miR-328-5p, a fragment of circ-GRB10 including the predicted target site or a mutated target site was constructed into the downstream part of the firefly luciferase gene (pGL3-circ-GRB10-Wild and pGL3-circ-GRB10-Mut). The plasmids were co-transfected with miR-328-5p mimics or NC into NP cells. miR-328-5p mimics induced a reduction in relative luciferase expression in pGL3-circ-GRB10-Wild compared with the NC. In contrast, there was no difference in the luciferase activity of pGL3-circ-GRB10-Mut between miR-328-5p mimics and the control (Fig. 4d). These data suggest that miR-328-5p directly targets circ-GRB10 in vitro.

### Prediction of miR-328-5p target genes

**Fig. 5:**
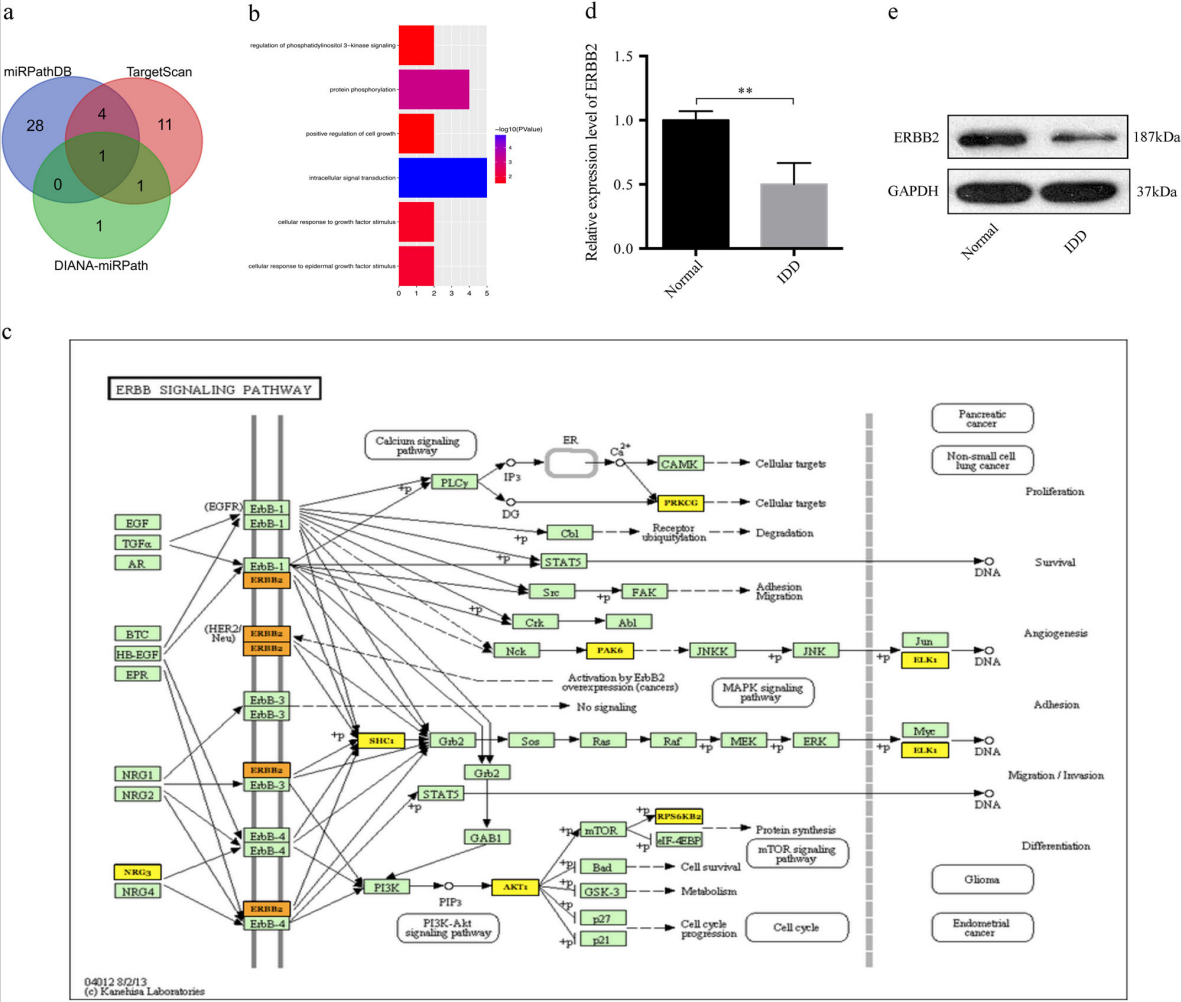
Prediction of miR-328-5p-mediated signaling pathways and function annotations for miR-328-5p. **a** The Venn diagram indicates the relevant pathways mediated by miR-328-5p. ErbB pathway was intersected predicted by three different databases. **b** DAVID function annotation for the miR-328-5p targeted genes of the ErbB pathways. The vertical axis shows the annotated functions of the target genes. The horizontal axis shows the gene number of each cluster respectively. The color indicated the −log10 transformed *P* value of each GO term. **c** Mapping of ErbB pathway mediated by miR-328-5p. In this map, circ-GRB10 could act as positive regulators of target genes, while miR-328-5p function as negative regulators. The yellow target genes could be regulated by the circ-GRB10/miR-328-5p axes to devote to the initiation and progression of IDD. **d**,** e** Expression of ERBB2 in IDD NP tissues, showing that ERBB2 expression was significantly lower than that of controls both at the mRNA **d** and protein **e** levels. **P < 0.01, *n* = 3

To investigate the functional effects of circ-GRB10/miR-328-5p, we predicted target pathways of miR-328-5p using the miRPathDB (https://omictools.com/), TargetScan (http://www.targetscan.org/), and DIANA-miRPath (http://diana.imis.athena-innovation.gr/) databases. The Venn diagram demonstrated that the ErbB signaling pathway was probably regulated by miR-328-5p (Fig. 5a). Eight genes (NRG3, ERBB2, SHC1, PRKCG, PAK6, AKT1, RPS6KB2, and ELK1) in the ErbB signaling pathway could be regulated by circ-GRB10/miR-328-5p. Then, DAVID functional annotation was performed for the target genes and revealed that these genes were significantly correlated with biological processes like protein phosphorylation, cellular response to growth factor stimulus, and positive regulation of cell growth (Fig. 5b). The data were integrated from the KEGG, miRPathDB, TargetScan, and DIANA-miRPath databases to draw the ErbB signaling network containing eight genes regulated by circ-GRB10/miR-328-5p (Fig. 5c), which indicated that ERBB2 is a possible target of miR-328-5p. The expression of ERBB2 in IDD NP tissues was assessed, and ERBB2 expression was significantly lower than that of controls (*P* < 0.01, Fig. 5d, e).

### Circ-GRB10 regulates the expression of ERBB2

**Fig. 6:**
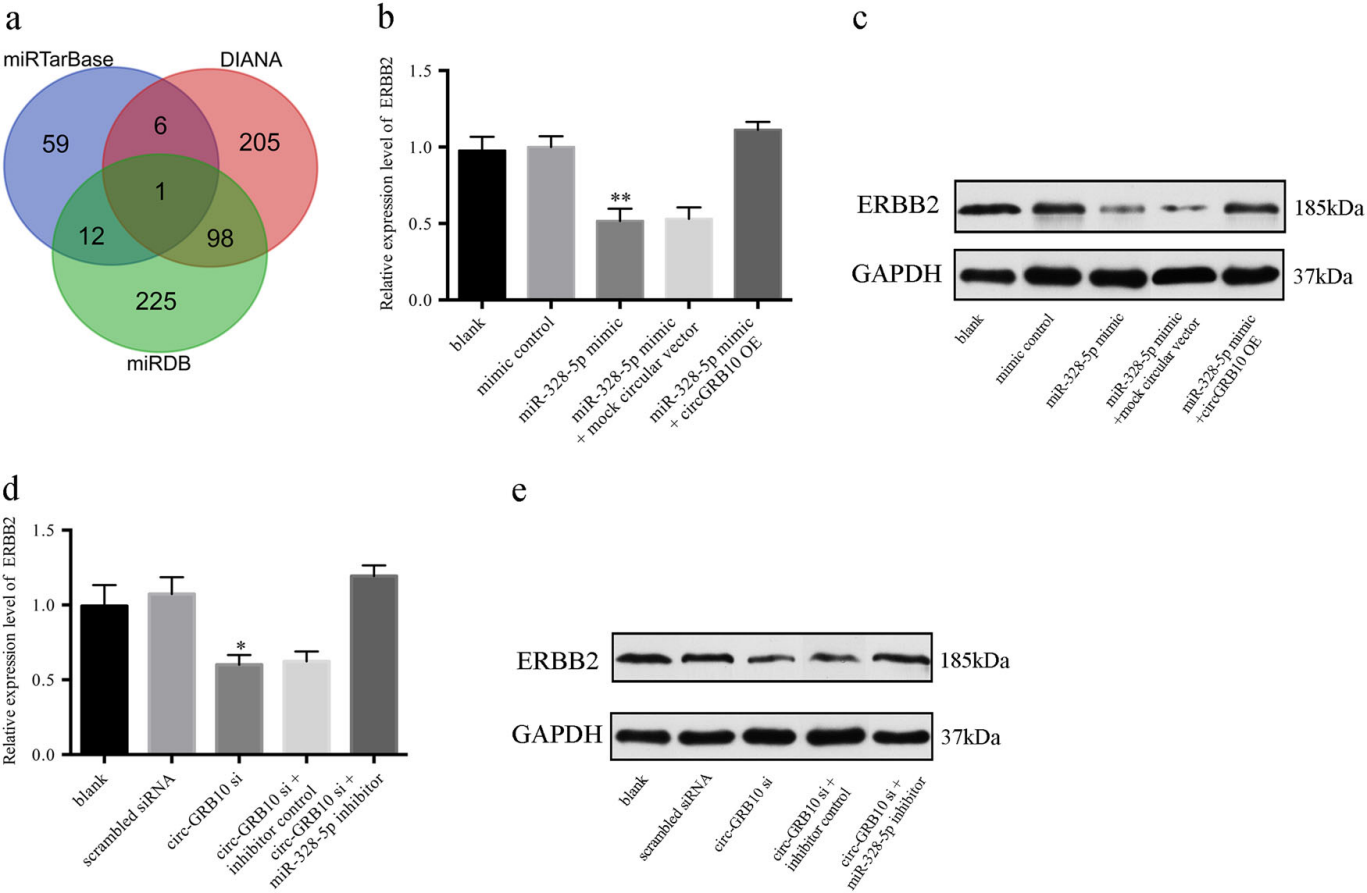
The circ-GRB10/miR-328-5p axis is critical for cell function. **a** The Venn diagram indicates the relevant target genes mediated by miR-328-5p. ERBB2 was intersected and predicted by three different databases. **b** NP cells from control tissues were transfected with miR-328-5p with or without circ-GRB10 overexpress plasmid. qRT-PCR was used to detect the relative mRNA levels of ERBB2 compared with controls. **c** Relative protein levels of ERBB2 when transfected with miR-328-5p mimics and reversed by circ-GRB10 expression plasmid. **d** circ-GRB10 siRNA with or without miR-328-5p inhibitor was transfected into NP cells from control tissues and the mRNA level of ERBB2 was evaluated by qRT-PCR. **e** Western blot analysis of ERBB2 protein level following treatment of NP cells from control tissues with circ-GRB10 siRNA or miR-328-5p inhibitor. GAPDH was used as control. *P < 0.05, **P < 0.01, *n* = 3

Bioinformatics prediction showed that ERBB2 was the target of miR-328-5p, as predicted by three microRNA target gene databases (DIANA, miRDB, and miRTarbase) (Fig. 6a). To determine whether circ-GRB10 could regulate ERBB2 expression through miR-328-5p, we analyzed the mRNA and protein levels of ERBB2 in NP cells isolated from normal tissues. The expression of circ-GRB10 was attenuated after siRNA silencing (*P* < 0.001) and increased by circ-GRB10 overexpression (*P* < 0.001) (Supplemental Fig. 3a). The expression of miR-328-5p was upregulated after miR-328-5p mimic addition (*P* < 0.001) and downregulated after miR-328-5p inhibitor treatment (*P* < 0.01) (Supplemental Fig. 3b). Moreover, the expression of miR-328-5p was altered after circ-GRB10 silencing or overexpression (Supplemental Fig. 3c). As shown in Fig. 6b, c, the expression of ERBB2 was inhibited by miR-328-5p mimics and could be reversed by circ-GRB10 overexpression at both the mRNA and protein levels. Circ-GRB10 silencing resulted in a reduction of ERBB2 at both the mRNA and protein levels, as well as in the downregulation of miR-328-5p. Meanwhile, the expression of ERBB2 was elevated in NP cells treated with a miR-328-5p inhibitor (Fig. 6d, e). These results indicated that circ-GRB10 regulates ERBB2 through miR-328-5p.

### Circ-GRB10-miR-328-5p-ERBB2 regulatory loop is critical for NP cells survival

**Fig. 7:**
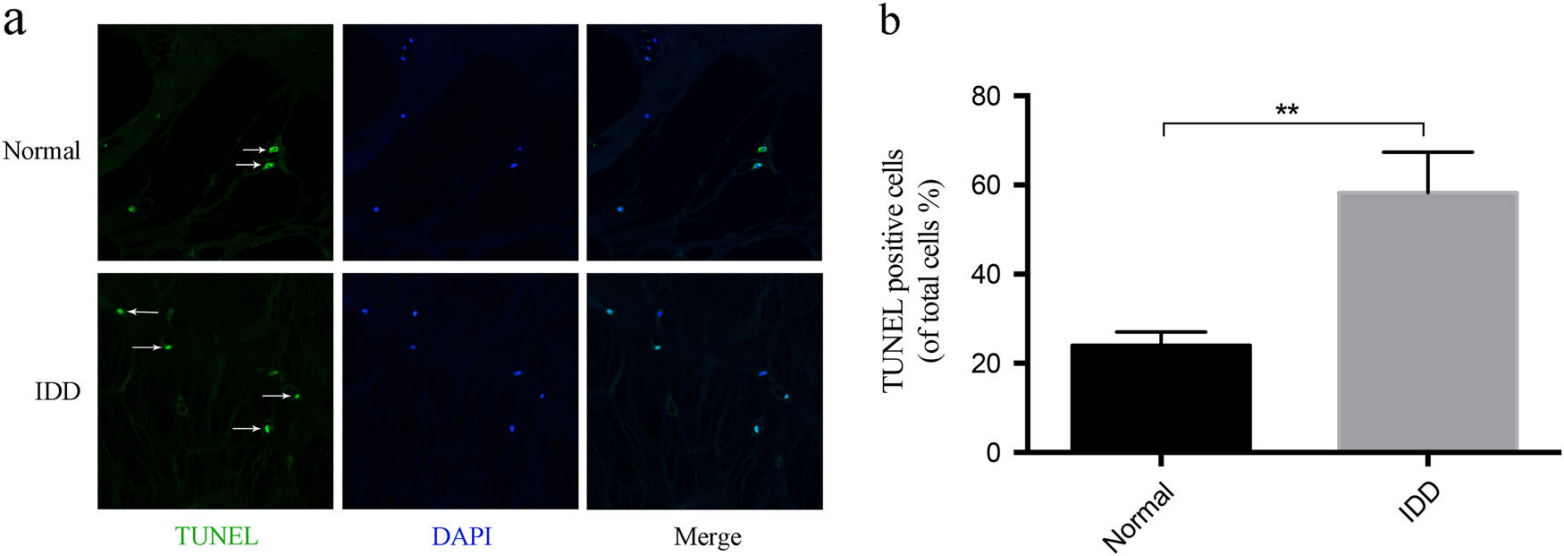
Apoptosis of IDD NP cells. **a** Tunel staining of normal and IDD NP tissue. **b** The cell death rate in IDD NP tissues was significantly higher than that in normal ones, as indicated by the TUNEL assay. **P < 0.01, *n* = 4

**Fig. 8:**
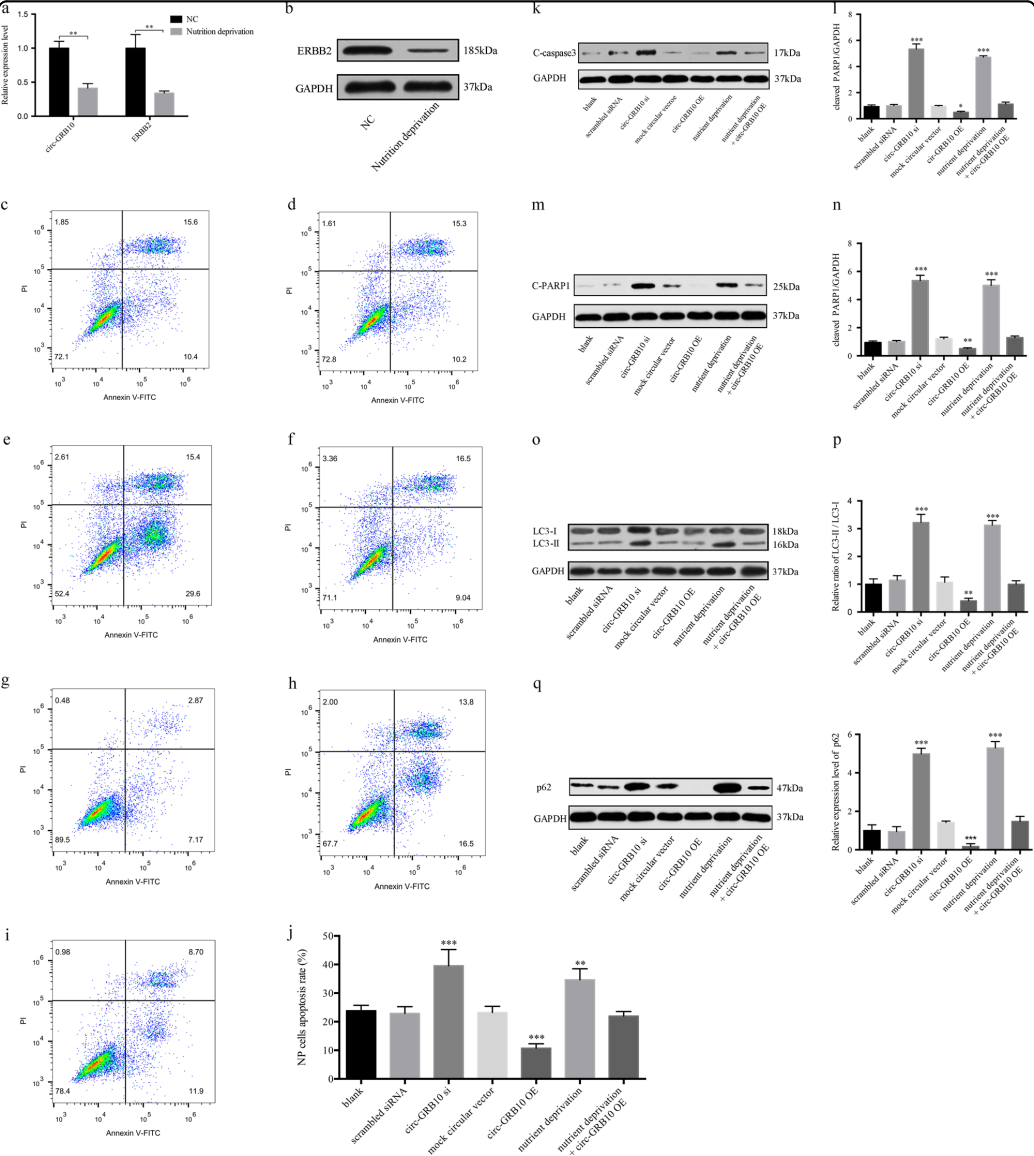
NP cells apoptosis rate in each group after transfection. **a**, **b** Constitutive expression levels of circ-GRB10 and ERBB2 protein in nutrient deprivation condition as well as normal ones. **c** Blank group. **d** Scrambled siRNA group. **e** circ-GRB10 si group. **f** Mock circular vector circRNA group. **g** circ-GRB10 overexpression (circ-GRB10 OE) group. **h** nutrient deprivation group. **i** Function recovery group, i.e., nutrient deprivation + circ-GRB10 OE group. **j** Apoptosis rate of each rate. **k**, **l** Expression of C-caspase3. **m**,** n** Expression of C-PARP1. **o**, **p** LC3-II/LC3-I ratios. **q**,** r** Expression of p62. (**P < 0.01, ***P < 0.001, *n* = 3. C-caspase3, cleaved caspase3; C-PARP1, cleaved PARP1)

As shown in Fig. 7a, b, the cell death rate in IDD NP tissues was significantly higher than in normal NP cells, as indicated by the TUNEL assay(*P* < 0.01). We next checked whether circ-GRB10 participated in NP cell apoptosis and survival. After nutrition deprivation, the constitutive expression of circ-GRB10 and ERBB2 was downregulated compared to normal cells (all *P* < 0.01, Fig. 8a, b). The apoptosis in NPs cells of the circ-GRB10 overexpression group was significantly suppressed compared with the mock circRNA vector group (*P* < 0.001); nevertheless, circ-GRB10 knockdown markedly increased the NP cell apoptosis rate (*P* < 0.001); circ-GRB10 could significantly stimulate NP cells survival under nutrient deprivation conditions (*P* < 0.01) (Fig. 8h). Moreover, the expression of apoptosis-related proteins was measured. As shown in Fig. 8k, i, the expression of cleaved caspase-3 was upregulated after circ-GRB10 silencing and by nutrient deprivation (both *P* < 0.001), and attenuated by circ-GRB10 overexpression (*P* < 0.05). Figure 8m, n shows that the expression of cleaved PARP1 was upregulated after circ-GRB10 silencing and by nutrient deprivation (both *P* < 0.001), and attenuated by circ-GRB10 overexpression (*P* < 0.01). The LC3-II/LC3-I ratio was increased after circ-GRB10 silencing and by nutrient deprivation (both *P* < 0.001), and decreased by circ-GRB10 overexpression (*P* < 0.01) (Fig. 8o, p). Moreover, Fig. 8q, r shows that the expression of p62 was upregulated after circ-GRB10 silencing and by nutrient deprivation (both *P* < 0.001), and attenuated by circ-GRB10 overexpression (*P* < 0.001).

## Discussion

The pathogenesis of IDD remains poorly understood. In this study, bioinformatics identified 104 differentially expressed circRNAs and 27 differentially expressed miRNAs in degenerative NP cells. Among them, circ-GRB10 could be a circRNA playing important roles in IDD. Dysregulated circ-GRB10 was a ceRNA-regulating ERBB2 expression by sponging miR-328-5p, which mediates the death of NP cells in IDD. Moreover, circ-GRB10 favored the survival of NP cells under nutrient deprivation conditions by upregulating the expression of ERBB2 in NP cells. These findings strongly suggest that circ-GRB10 play an essential role in the pathogenesis of IDD.

Intervertebral disc degeneration changes are clinically important as they are associated with back pain and morbidity. Cell death is a fundamental biological process that can be separated into three classes: apoptosis, autophagy, and necrosis^26^. The deregulation of cell death is associated with the etiology, pathogenesis, and treatment of many diseases^18,27,28^ such as cancer, Alzheimer’s disease, heart diseases, and Parkinson’s disease^29,30^. Over the past years, increasing evidence has indicated that NP cell death contributes to spinal degenerative diseases and IDD^7,10,31^.

The intervertebral disc is avascular and the NP cells depend on diffusion from blood vessels at the disc’s margins to supply the nutrients essential for cellular activity and viability and to remove metabolic wastes^32^. Loss of nutrient supply can lead to NP cell death, loss of matrix production, and matrix degradation, and consequently to disc degeneration^32^. Nutrient deprivation can increase autophagy, which promotes apoptosis or lead to “autophagic cell death”^32–34^. In addition to nutrient deprivation, the regulation of autophagy involves multiple pathways and cellular stresses^35^. Autophagy itself is a protective process through which the cells recycle cellular components to ensure their survival, but excessive autophagy triggers the apoptosis of NP cells under conditions of nutrient deprivation^34^. In addition, nutrient deprivation may induce autophagosome formation^36^ and NP cell autophagy by increasing the ratios of LC3-II/LC3-I and beclin-1/β-actin^34^. Two well-characterized signaling cascades, including the target of rapamycin (TOR) and Ras-cAMP-dependent protein kinase A (PKA) pathways, sense nutrient status^35^. TOR regulates nutrient sensing, cell growth, and autophagy^37^. TOR activates downstream proteins, including Akt kinase (also known as protein kinase B), phosphoinositide-3 kinase (PI3K), and growth factor receptor^38^. The inhibition of autophagy significantly decreases the rate of apoptosis in cells disrupted by H_2_O_2_^39^. Hence, controlling the autophagy response in NP cells could enhance cell survival and probably delays disc degeneration.

The present study showed that circ-GRB10 overexpression significantly decreased apoptosis of NP cells under nutrient deprivation environment, suggesting that circ-GRB10 is beneficial for NP cells survival under nutrient deprivation conditions. In addition, the results suggested that apoptosis and autophagy are also involved in the pathogenesis of IDD, as indicated by alterations of cleaved caspase3, cleaved PARP1, LC3II/LC3I, and p62. Accordingly, circ-GRB10 knockdown increased the apoptosis rate of NP cells, and circ-GRB10 overexpression inhibited the death of NP cells in vitro.

A previous study demonstrated that ERBB2 inhibits autophagy via the formation of a complex with Beclin 1, which is a key regulator of autophagy^40^. In addition, lapatinib, a dual tyrosine kinase inhibitor of EGFR and ERBB2, induced autophagic cell death in breast cancer cells overexpressing ERBB2^40^, human hepatoma cells^41^, and acute myeloblastic leukemia^42^. In the present study, circ-GRB10 could regulate ERBB2 expression by sponging miR-328-5p. These findings indicated that dysregulation of circ-GRB10 may contribute to IDD progression by regulating NP cell apoptosis/survival. This could be a potentially novel therapeutic target for IDD.

Unfortunately, whether these results are due to circ-GRB10 acting as a sponge, to circ-GRB10 enhancing the degradation of miR-328-5p, or to decreased miR-328-5p expression remains to be elucidated. To do so, co-expression of both the circRNA and miRNA will be performed in the future^43^. Nevertheless, the Pearson correlation analysis showed a fairly good anti-correlation between circ-GRB10 and miR-328-5p, highlighting their relationship but without revealing its nature. In addition, the FISH analysis revealed that both were localized in the same cellular compartment (cytoplasm), further supporting this correlation.

In conclusion, the present study demonstrated that circ-GRB10 facilitated the survival of NP cells during nutrient deprivation by upregulating ERBB2 expression through inactivating miR-328-5p, and possibly through a pathway involving mTOR. These findings may be valuable to better understand the molecular mechanisms involving the survival and death of the intervertebral disk cells, and suggested a promising therapeutic target for IDD treatment.

## Materials and methods

### Ethics statement

This study was approved and supervised by the Ethics Committee of Tianjin Medical University General Hospital and Hebei Province Cangzhou Hospital of Integrated Traditional and Western Medicine. Human NP tissues were obtained from patients undergoing surgery at The Tianjin Medical University General Hospital and Hebei Province Cangzhou Hospital of Integrated Traditional and Western Medicine. Written informed consents were obtained from all patients for the use of their tissues for research purposes.

### Clinical specimens

Human lumbar degenerative NP specimens were obtained from 20 patients with IDD undergoing discectomy. The control samples were taken from 20 age- and sex-matched patients with fresh traumatic lumbar fracture who underwent anterior decompressive surgery because of neurological deficits. Table 2 presents the characteristics of the patients.

**Table 2:** Clinical features of study population

Variable	Normal (*n* = 20)	IDD (*n* = 20)	*P*
Age (years, mean, SD)	37.75 (8.47)	41.15 (11.19)	0.286^a^
BMI (kg m^2^)	24.08 (1.82)	25.06 (2.15)	0.129^a^
*Sex (%)*			
Male	13 (65)	14 (70)	0.736^b^
Female	7 (35)	6 (30)	

^a^Student’s *t* test

^b^Two-sided *χ*^2^-test

### Microarray data

CircRNA expression data set (GSE67566) and miRNA expression data set (GSE63492) were downloaded from the Gene Expression Omnibus database (http://www.ncbi.nlm.nih.gov/geo)^16^. There were five human NP samples derived from patients with IDD and five samples derived from cadaveric disc as normal control. The platform was the GPL19978 Agilent-069978 Arraystar Human CircRNA microarray V1 for circRNA and GPL19449 Exiqon miRCURY LNA microRNA Array for miRNA. Probe annotation files were also acquired.

### Preprocessing and differential analysis

Raw data were converted into recognizable format with the package affy of R. Missing values were imputed by a method based on the K nearest neighbors (KNNs). The KNN-based method selects genes with expression profiles similar to the gene of interest to impute missing values^44^. After background correction and data normalization with the median method^45^, differential analysis was performed using the limma package between degeneration samples and controls. The first is the design matrix, which indicates which RNA samples have been applied to each array. The second is the contrast matrix, which specifies which comparisons you would like to make between the RNA samples. For statistical analysis and assessing differential expression, limma uses an empirical Bayesian method to moderate the standard errors of the estimated log-fold changes. The basic statistic used for significance analysis is the moderated *t*-statistic, which is computed for each probe and for each contrast. Moderated *t*-statistics lead to *P* values in the same way that ordinary *t*-statistics do, except that the degrees of freedom are increased to reflect the greater reliability associated with the smoothed standard errors. Limma provides the topTable and decideTests functions, which summarize the results of the linear model, perform hypothesis tests, and adjust the *P* values for multiple testing. Results include (log) fold changes, standard errors, *t*-statistics, and *P* values^46^. |Log(fold change)| >2 and adj. *P* value <0.05 were set as the cutoffs to identify differentially expressed RNAs.

### Annotation for circRNA–miRNA interaction

The circRNA–miRNA interactions were predicted using miRNA target prediction based on the TargetScan^47^ and miRanda^48^ databases. To establish the circRNA–miRNA network, we searched MREs on circRNAs, then selected the miRNAs according to the seed match sequences. The interaction networks between circRNAs and predicted miRNAs were then visualized with Cytoscape.

### Quantitative real-time RT-PCR

After RNA extraction, the M-MLV reverse transcriptase (Invitrogen, Carlsbad, CA) was used for complementary DNA (cDNA) synthesis, according to the manufacturer’s instructions. The expression of circ-GRB10 was evaluated by quantitative PCR using the SYBR Green assay. Specific divergent primers were designed to amplify the circular transcripts (forward: 5′-GCCGCCGCAAAGCAGATATTC-3′; reverse: 5′-ACAGACTCCAGCAGGGTCAG-3′). All primers used in this study are listed in Table 3. PCR was performed in a 10-μL reaction volume, including 2 μL of cDNA, 5 μL of 2× Master Mix, 0.5 μL of forward primer (10 μM), 0.5 μL of reverse primer (10 μM), and 2 μL of double-distilled water. The reaction was set at 95 °C for 10 min for pre-denaturation, then at 95 °C for 10 s and at 60 °C for 60 s, repeated for 40 cycles. GAPDH was used as a reference. The target and reference were amplified in triplicate. The relative expression of each circRNA was calculated using the 2^−△△Ct^ method^49^.

**Table 3:** Sequences of primers for qRT-PCR and siRNA-related sequence

Name		Sequence
circ-GRB10	Forward	5′-GCCGCCGCAAAGCAGATATTC-3′
	Reverse	5′- ACAGACTCCAGCAGGGTCAG-3′
miR-328-5p	Forward	5′-ACACTCCAGCTGGGGGGGGGGCAGGAGGGGC-3′
	Reverse	5′-CTCAACTGGTGTCGTGGA-3′
U6	Forward	5′-CTCGCTTCGGCAGCACA-3′
	Reverse	5′-AACGCTTCACGAATTTGCGT-3′
ERBB2	Forward	5′-TGTGACTGCCTGTCCCTACAA-3′
	Reverse	5′-CCAGACCATAGCACACTCGG-3′
GAPDH	Forward	5′-GCACCGTCAAGGCTGAGAAC-3′
	Reverse	5′-GGATCTCGCTCCTGGAAGATG-3′
circ-GRB10 si		5′-AGCAGATATTCTGGAGGAA-3′
miR-328-5p mimics	Sense	5′-GGGGGGGCAGGAGGGGCUCAGGG-3′

### Isolation and culture of human NP cells

Tissue specimens were washed twice with phosphate-buffered saline (PBS). Nucleus pulposus was separated from the annulus fibrosus using a stereotactic microscope and cut into pieces (2–3 mm^3^). Nucleus pulposus cells were released from tissues by incubation with 0.25 mg/mL of type II collagenase (Invitrogen, Carlsbad, CA) for 12 h at 37 °C in Dulbecco’s modified Eagle medium (DMEM/F12; GIBCO, Grand Island, NY). After isolation, NP cells were resuspended in DMEM/F12 containing 10% FBS (GIBCO), 100 mg/mL streptomycin, 100 U/mL penicillin, and 1% l-glutamine, and then incubated at 37 °C in a humidified atmosphere with 5% CO_2_. The confluent cells were detached by trypsinization, seeded into 35-mm tissue culture dishes in complete culture medium (DMEM/F12 supplemented with 10% FBS, 100 mg/mL streptomycin, and 100 U/mL penicillin) in a 37 °C, 5% CO_2_ environment. The medium was changed every 2 days. The cells at the second passage were used for subsequent experiments.

### siRNA and circ-GRB10 overexpression plasmid construction

According to the circRNA sequences of circ-GRB10 (hsa_circ_0080210) in circBase, the siRNA of circ-GRB10 (each siRNA had three pairs of sequences) and negative controls (NCs) were designed and synthesized by Guangzhou Geenseed Biotech Co., Guangzhou, China. To induce GRB10 transcript formation in vitro by nonlinear splicing, the circ-GRB10 overexpression vector was constructed. The front and back circular frames were synthesized and added to pLCDH-ciR for the circularization of the transcripts. As previously reported, the front circular frame contains the endogenous flanking genomic sequence with the EcoR I restriction site, and the back circular frame contains part of the inverted upstream sequence with the BamH I restriction site^50^. The cDNA encoding circ-GRB10 in Hela cells was amplified using the primers forward: cgGAATTCTGAAATATGCTATCTTACAGATATTCTGGAGGAAGGTGTGA and reverse: cgGGATCCTCAAGAAAAAATATATTCACCTGCTTTGCGGCGGCCTGGCTCGGAGGTAA. The 637-bp target fragment contains, in order, the EcoR I site, splice acceptor AG, circ-GRB10 sequence, splice donor GT, and BamH I site. Then, the amplified fragment was cloned into the vector between the two frames. We also established a mock vector only containing a non-sense sequence between the two circular frames without the circ-GRB10 cDNA. Vector construction was verified by direct sequencing. The vectors were constructed with the help of Guangzhou Geenseed Biotech Co., Guangzhou, China.

### Fluorescence in situ hybridization

Fluorescence in situ hybridization was performed to detect subcellular location of circ-GRB10 and miR-328-5p according to the method described by Vautrot^51^. A FISH probe labeled with Alexa Fluor(^®^) 488 for circ-GRB10 was designed to detect the splicing junction of two exons. The probe sequence was 5′-AGTCAAAGCGAATGTCAAGTGTCTGGCACCTCCC-3′. The probe for miR-328-5p was labeled with cy3; the probe sequence was 5′-CCCTGAGCCCCTCCTGCCCC C-3.

### Cell transfection

The third-generation NP cells were used for transfection. Culture plates were incubated at 37 °C in a humidified atmosphere with 5% CO_2_. Cells were transfected with corresponding plasmids or siRNAs using Lipofectamine 3000 (Invitrogen, Carlsbad, CA), according to the manufacturer’s recommendations. The cells were collected 48 h after transfection.

### Dual-luciferase reporter assay

The binding site of circ-GRB10 (either wild type or mutated) were inserted into the KpnI and SacI sites of the pGL3 promoter vector (Realgene, Nanjing, China) in the dual-luciferase reporter assay. Firstly, cells were plated on 24-well plates. Then, 80 ng of plasmid, 5 ng of renilla luciferase vector pRL-SV40, 50 nM miR-328-5p mimics, and NCs were transfected into the cells using lipofectamine 3000 (Invitrogen, Shanghai, China). Cells were collected and measured according to the manufacturer’s instructions using the Dual-Luciferase Assay (Promega, Madison, WI, USA) after 48 h of transfection. All experiments were repeated three times independently.

### Western blotting

Cultured cells were homogenized in lysis buffer (0.25 M Tris-HCl, pH 6.8, 20% glycerol, 4% SDS, 10% mercaptoethanol) supplemented with protease and phosphatase inhibitors. Samples containing equal amounts of protein (10 µg) were separated in SDS-polyacrylamide gels (SDS-PAGE, 10–12%). Proteins were transferred to polyvinylidene fluoride membranes. The membranes were blocked with 5% non-fat milk in Tris-buffered saline containing 0.1% Tween-20 (TBST) at room temperature for 1 h. The membranes were incubated overnight at 4 °C with primary antibodies (1:3000) in TBST containing 5% non-fat milk. The secondary antibodies (1:6000) were incubated at room temperature for 1 h. The immunoblots were developed using an ECL system.

### Flow cytometry

Nucleus pulposus cells were assigned to the NC group (cells transfected with empty vector and cultured with 10% FBS), circ-GRB10 si group (cells transfected with siRNA-circ-GRB10 and cultured with 10% FBS), circ-GRB10 overexpression (circ-GRB10 OE) group (cells transfected with pLCDH-cir-GRB10 and cultured with 10% FBS), nutrient deprivation group (cells transfected with empty vector and cultured with DMEM/F12), and function recovery group (cells transfected with pLCDH-cir-GRB10 and cultured with DMEM/F12).

After transfection for 48 h, the medium was discarded and the cells were washed with PBS and digested with 0.25% trypsin. PBS was used to resuspend the cells, followed by centrifugation at 2000 r.p.m. per min for 5 min. The precipitate was washed twice with PBS and cell concentration was adjusted to 1 × 10^6^ cells per mL. Cells were fixed with precooled 70% alcohol for 30 min, centrifuged, and stained with propidium iodide (PI) containing RNA enzyme (GR1-25, SBS Genetech, Beijing, China) for 30 min. The samples were detected using a flow cytometer (FACS Calibur, Becton, Dickinson and Company, New Jersey, USA) and recorded at excitation wavelength of 488 nm.

Cells in the logarithmic growth phase were digested with 0.25% trypsin. Cells were washed three times with cool PBS, centrifuged, and resuspended with buffer. According to the instruction of the Annexin V-FITC/PI apoptosis kit (Abnova, Walnut, CA, USA), 5 μL of Annexin-V-FITC, and 50 μL of PI were added for reaction at 4 °C for 15 min. Then, 300 μL of binding buffer were added. Cell apoptosis was detected at excitation wavelength of 488 nm and emission wavelengths of 515 and 560 nm. The experiments were repeated three times and the average value was taken.

### TUNNEL assay

Deparaffinization was achieved by serial gradient ethanol. Antigen retrieval was carried out using Proteinase K (Beyotime, China). Then, 0.3% Triton X-100 was used to permeated cell membrane. TdT and dUTP (2:29) were incubated at room temperature for 2 h. The cells were counter-stained with DAPI to visualize the nuclei under a fluorescence microscope (Olympus, Tokyo, Japan).

### Statistical analysis

Each experiment was repeated at least three independent times, and the cells in each experiment were collected from a single isolation. The results of real-time quantitative RT-PCR and the gray values from western blotting are shown as mean ± SEM. Statistical significance was determined using a two-tailed Student’s *t* test (SPSS 22.0). Significance was set at *P < 0.05, **P < 0.01, ***P < 0.001.

## Electronic supplementary material

Supplemental Figure 1

Supplemental Figure 2

Supplemental Figure 3

Table S1

Table S2

Supplementary Information
