# Supplementary figures and images for: Circular RNA GRB10 as a competitive endogenous RNA regulating nucleus pulposus cells death in degenerative intervertebral disk
Source: Cell Death Dis. 2018 Feb 23;9(3):319. doi: 10.1038/s41419-017-0232-z (PMC5833826; doi:10.1038/s41419-017-0232-z)

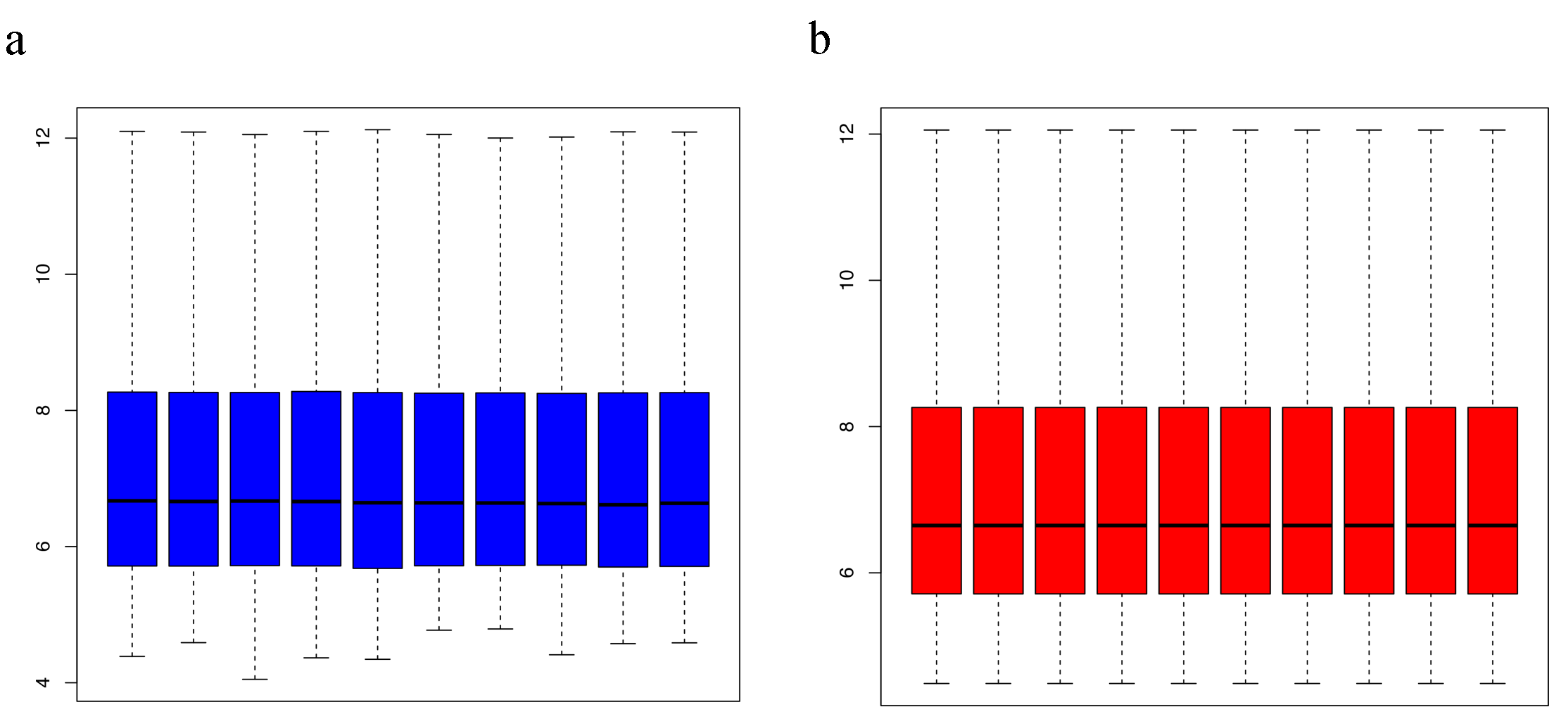

Supplement: Supplementary file 1 — Supplemental Figure 1 [file 41419_2017_232_MOESM1_ESM.tif]

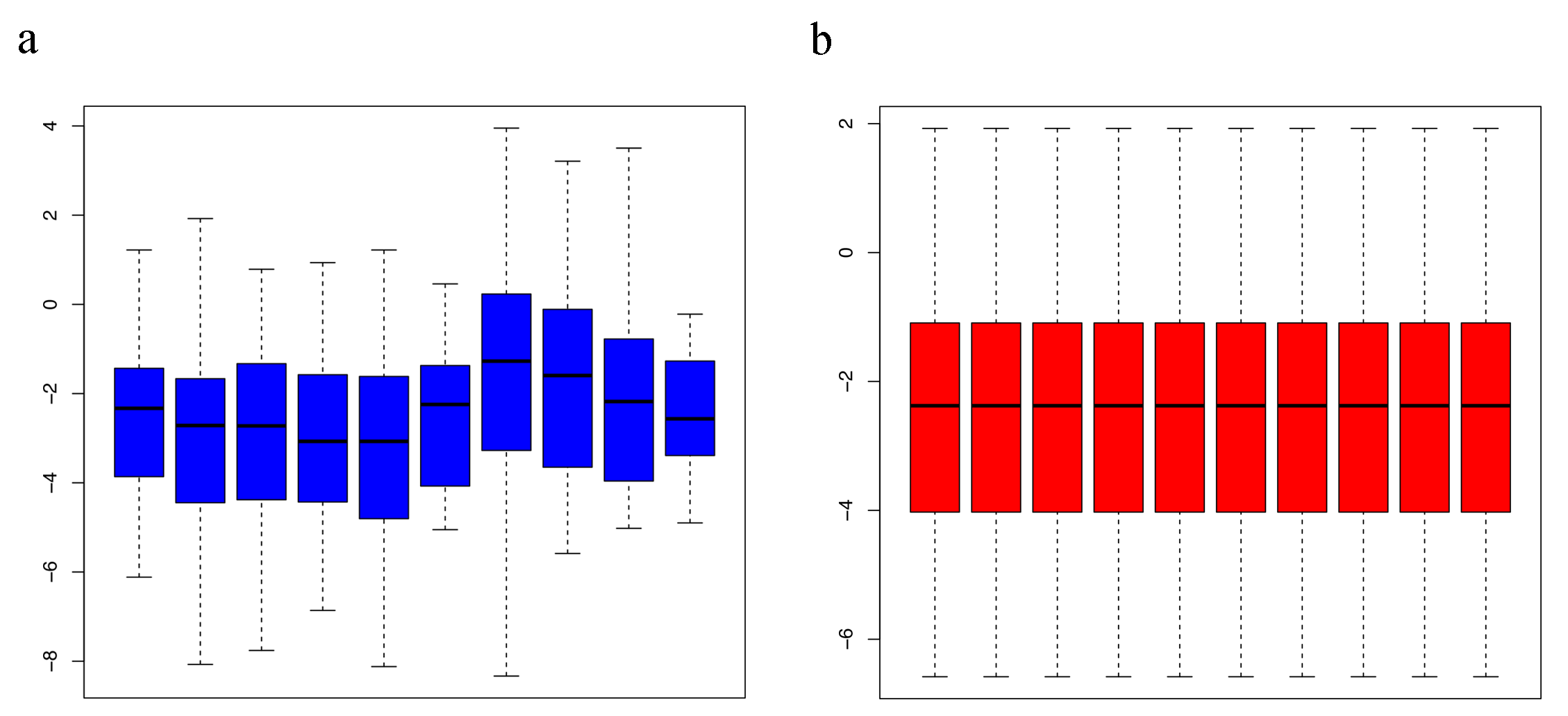

Supplement: Supplementary file 2 — Supplemental Figure 2 [file 41419_2017_232_MOESM2_ESM.tif]

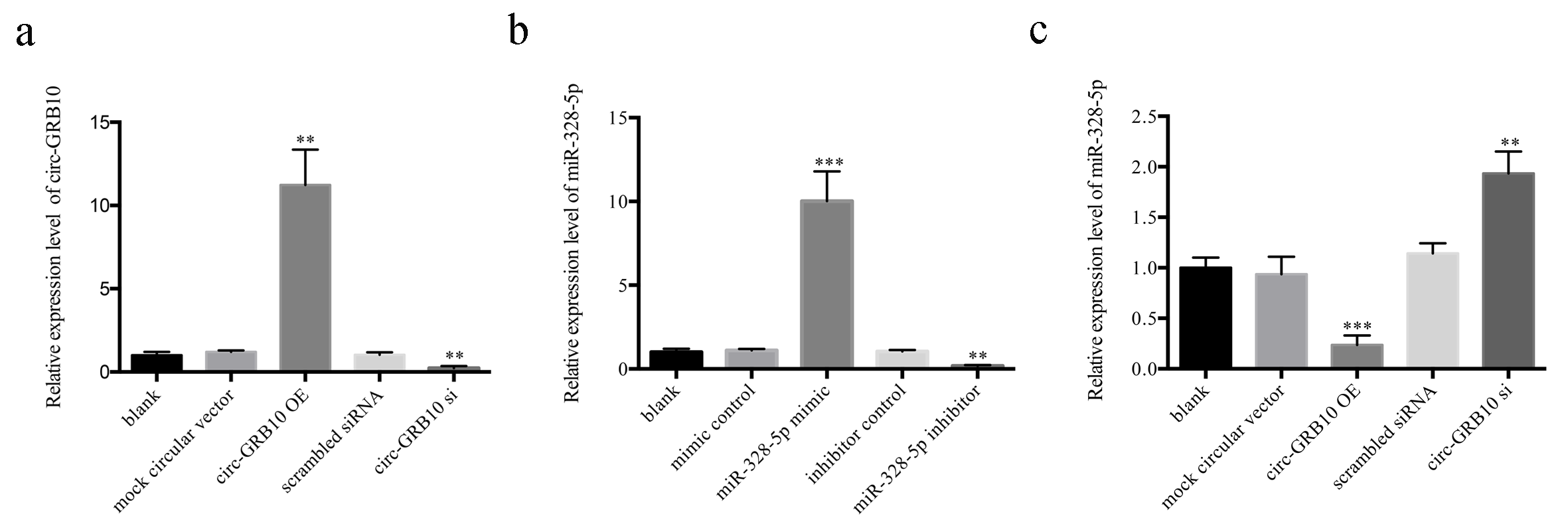

Supplement: Supplementary file 3 — Supplemental Figure 3 [file 41419_2017_232_MOESM3_ESM.tif]
