# Supplementary material for: Circular RNA GRB10 as a competitive endogenous RNA regulating nucleus pulposus cells death in degenerative intervertebral disk
Source: Cell Death Dis. 2018 Feb 23;9(3):319. doi: 10.1038/s41419-017-0232-z (PMC5833826; doi:10.1038/s41419-017-0232-z)
Supplement: Supplementary file 6 — Supplementary Information [file 41419_2017_232_MOESM6_ESM.docx]

**Supplemental figure legends**

**Figure S1. Box plot of circRNAs expression data.** (a) Represent the circRNAs date before normalization. (b) Represent the circRNAs date after normalization. The medians (black lines) are almost at the same level, indicating a good performance of normalization.

**Figure S2. Box plot for miRNAs expression data.** (a) Represent the miRNAs date before normalization. (b) Represent the miRNAs date after normalization. The medians (black lines) are almost at the same level, indicating a good performance of normalization.

**Figure S3.** (a) The relative expression levels of circ-GRB10 were markedly increased by circ-GRB10 OE compared with controls, while circ-GRB10 siRNA significantly decreased the expression of circ-GRB10. (b) The relative expression levels of miR-328-5p were markedly increased by miR-328-5p mimic compared with controls, while the miR-328-5p inhibitor significantly decreased the expression of miR-328-5p. (c) The relative expression levels of miR-328-5p were markedly decreased by circ-GRB10 OE compared with controls, while the circ-GRB10 siRNA significantly increased the expression of miR-328-5p. ** P < 0.01, *** P < 0.001, n=3.
